# N-type voltage gated calcium channels mediate excitatory synaptic transmission in the anterior cingulate cortex of adult mice

**DOI:** 10.1186/1744-8069-9-58

**Published:** 2013-11-14

**Authors:** SukJae Joshua Kang, Ming-Gang Liu, Tian-Yao Shi, Ming-Gao Zhao, Bong-Kiun Kaang, Min Zhuo

**Affiliations:** 1 Department of Brain and Cognitive Sciences, College of Natural Sciences, Seoul National University, Seoul 151-746, South Korea; 2 Department of Physiology, Faculty of Medicine, University of Toronto, 1 King's College Circle, Toronto, Ontario M5S 1A8, Canada; 3 Department of Pharmacology, School of Pharmacy, Fourth Military Medical University, Xi'an 710032, China; 4 Department of Biological Sciences, College of Natural Sciences, National Creative Research Initiative Center for Memory, Seoul National University, Seoul 151-747, South Korea; 5 Center for Neuron and Disease, Frontier Institute of Science and Technology, Xi’an Jiaotong University, Xi’an 710049, China

## Abstract

Voltage gated calcium channels (VGCCs) are well known for its importance in synaptic transmission in the peripheral and central nervous system. However, the role of different VGCCs in the anterior cingulate cortex (ACC) has not been studied. Here, we use a multi-electrode array recording system (MED64) to study the contribution of different types of calcium channels in glutamatergic excitatory synaptic transmission in the ACC. We found that only the N-type calcium channel blocker ω-conotoxin-GVIA (ω-Ctx-GVIA) produced a great inhibition of basal synaptic transmission, especially in the superficial layer. Other calcium channel blockers that act on L-, P/Q-, R-, and T-type had no effect. We also tested the effects of several neuromodulators with or without ω-Ctx-GVIA. We found that N-type VGCC contributed partially to (1S,3R)-1-aminocyclopentane-1,3-dicarboxylic acid- and (R)-Baclofen-induced synaptic inhibition. By contrast, the inhibitory effects of 2-Chloroadenosine and carbamoylcholine chloride did not differ with or without ω-Ctx-GVIA, indicating that they may act through other mechanisms. Our results provide strong evidence that N-type VGCCs mediate fast synaptic transmission in the ACC.

## Background

It is well-known that voltage-gated Ca^2+^ channels (VGCCs) play pivotal roles in neurotransmitter release and synaptic transmission. Previous studies have discovered the role of various types of calcium channels in peripheral regions [1], spinal cord [2], cerebellum [3] and hippocampus [4-6]. These studies indicate that N- (Ca_V2.2_) and P/Q-type (Ca_V2.1_) VGCCs play the most dominant role in basal synaptic transmission in most of the neurons [7,8]. N-type is more important in the peripheral nervous system and the joint action of N- and P/Q-type is prominent in the central nervous system [9,10]. These studies were performed by using the ω-conotoxin GVIA (ω-Ctx GVIA) and ω-agatoxin IVA (ω-Aga IVA), which specifically block the N- and P/Q-type VGCCs, respectively.

Due to its important role in neuronal Ca^2+^ concentration regulation, VGCCs are crucial players in a range of physiological and pathological conditions including acute nociception and chronic pain [11-13]. Among different VGCCs, N- and T- type VGCCs are known to play major roles in pain information processing [14,15] and inhibiting VGCCs is thought to be useful for reducing pain [16-18]. Ziconotide (SNX111; Prialt), a drug that targets N-type VGCC approved by the US Food and Drug Administration and European Medicine Agency, is also used intrathecally for severe chronic pain patients [18-21]. However, fewer studies have been reported for the role of VGCCs in synaptic transmission in pain-related cortical structures.

Convergent evidences from human and animal studies show that neurons in the anterior cingulate cortex (ACC) play important roles in pain perception and chronic pain [22,23]. Our previous studies show that neuropathic pain models induced long-term changes in excitatory synaptic transmission in the ACC neurons of adult mice [24,25]. Inactivation of the frontal cortex, including the ACC, by local lesions leads to the reduction of the nociceptive responses and aversive behaviors associated with chronic pain [26-29]. *In situ* hybridization brain atlas from the Allen Institute for Brain Science shows that N-, P/Q-, L-, T-, and R-type VGCCs are all expressed in the mouse ACC. Thus, in the present study, we used a 64-channel multi-electrode dish (MED64) system, a two-dimensional electrical activity monitoring device [30-32], to characterize the role of different VGCCs in adult mouse ACC glutamatergic synaptic transmission. The MED64 system allowed us to detect the field excitatory postsynaptic potentials (fEPSPs) at multiple sites in the mouse ACC at the same time, which is difficult to achieve with conventional field recording systems [24,30]. We found that N-type VGCCs play the dominant role in the ACC synaptic transmission and other VGCCs such as P/Q-, L-, T-, and R-type do not play any important role. Moreover, excitatory synaptic transmission in the ACC is subjected to strong and elegant modulation by various neuromodulators.

## Materials and methods

### Animals

Adult (8–12 week old) male C57BL/6 mice were used. All animals were housed under a 12 h light/dark cycle with food and water provided *ad libitum*. All works were conducted according to the policy and regulation for the care and use of laboratory animals approved by Institutional Animal Care and Use Committee in Seoul National University and University of Toronto. The number of animals used and their suffering were greatly minimized.

### Brain slice preparation

The general procedures for making ACC slices are similar to those described previously [30]. Briefly, adult male mice were anesthetized with isoflorane and the brains were removed and transferred to ice-cold artificial cerebrospinal fluid (ACSF) containing (in mM): 124 NaCl, 2.5 KCl, 2 CaCl_2_, 2 MgSO_4_, 25 NaHCO_3_, 1 NaH_2_PO_4_, 10 glucose. This ACSF was used throughout the experiment. Three coronal brain slices (300 μm), after the corpus callosum meets, were cut using a vibratome (Leica, Hesse, Germany). The slices were placed in a submerged recovery chamber with oxygenated (95% O_2_, 5% CO_2_) ACSF at room temperature for at least 2 h.

### Preparation of the multi-electrode array

The procedures for preparation of the MED64 system (Panasonic, Osaka, Japan) were similar to those as previously described [30-33]. The MED64 probe (MED-P515A, 8 x 8 array, interpolar distance 150 μm, Panasonic) was superfused with ACSF (pH = 7.4) at 28–30°C, and maintained at a 1 ~ 2 ml/min flow rate. One planar microelectrode with bipolar constant current pulses (1–20 μA, 0.2 ms) was used for stimulation of the ACC slice. The stimulation site was selected within the layer V region. Before use, the surface of the MED64 probe was treated with 0.1% polyethyleneimine (Sigma, St. Louis, MO, USA) in 25 mM borate buffer (pH 8.4) overnight, at room temperature.

### Field potential recording in adult ACC slices

After 2 h recovery, one ACC slice was placed in a MED probe, and most of the 64 electrodes located within the ACC. The slice was allowed to recover for 30 min before the electrophysiological recording was attempted. Electrical stimulation was delivered to one channel located within the layer V of the ACC, and evoked fEPSPs were monitored and recorded from the other 63 channels as described previously [24,30]. The intensity of the stimuli was approximately 40 ~ 60% of the intensity that induced the maximal fEPSPs. Baseline responses were evoked at 0.017 Hz for 30 min. The data were averaged every 2 min.

### Whole-cell patch-clamp electrophysiology

For whole-cell patch-clamp electrophysiology, slices were individually transferred to a recording chamber on the stage of a BX51WI microscope (Olympus, Tokyo, Japan) equipped with infrared differential interference contrast optics and superfused with the same ACSF at 2 ml/min for visualized whole-cell patch-clamp recordings [34-37]. Excitatory postsynaptic currents (EPSCs) were recorded from superficial layer (layer II-III) pyramidal neurons with an Axon 200B amplifier (Axon Instruments, Union City, CA, USA) and the stimulations were delivered by a bipolar tungsten-stimulating electrode placed in the deep layer (layer V-VI). AMPA receptor-mediated EPSCs were induced by repetitive stimulations at 0.03 Hz, and neurons were voltage clamped at −70 mV. The recording pipettes (borosilicate glass, 3–5 MΩ) were filled with a solution containing (in mM) 145 K-gluconate, 5 NaCl, 1 MgCl2,0.2 EGTA, 2 Mg-ATP, 0.1 Na3-GTP, 10 HEPES (adjusted to pH 7.2 with KOH; 280–300 mOsm). The initial access resistance (typically 15–30 MΩ) was monitored throughout the experiment. Data were discarded if the access resistance changed >15% during an experiment. Data were filtered at 1 kHz, and digitized at 10 kHz.

### Drugs

Drugs were freshly prepared: N-type VGCC blocker ω-Ctx GVIA, P/Q-type VGCC blocker ω-Aga IVA and R-type VGCC blocker SNX-482 were purchased from Peptide Institute Inc. (Osaka, Japan). T-type VGCC blocker NiCl_2_ was obtained from Sigma. L-type VGCC blocker nimodipine, metabotropic glutamate receptor (mGluR) agonist (1S,3R)-1-aminocyclopentane-1,3-dicarboxylic acid (1S,3R-ACPD), cholinergic receptor agonist carbamoylcholine chloride (carbachol, Cch), γ-Aminobutyric acid B (GABA_B_) receptor agonist (R)-Baclofen and adenosine receptor agonist 2-Chloroadenosine (2-CA) were purchased from Tocris Bioscience (Bristol, UK). Drugs were stored in frozen aliquots at −20°C. All drugs were diluted from the stock solutions to the final desired concentration in the ACSF before immediate use.

### Data analysis

MED64 Mobius was used for data acquisition and analysis. The percentages of the fEPSP slopes were normalized by the averaged value of the baseline period (30 min). The reduction index is calculated as follows: 100% - the percentage change of fEPSP slope within the last 4 min of the experiment. The value used for ‘end of drug treatment’ was the averaged value of interval between 4 min before and 2 min after endpoint. Whole-cell patch-clamp data were collected and analyzed using Clampex 10.2 and Clampfit10.2 software (Axon Instruments). All data are presented as mean ± SEM. Statistical comparisons were made using the t-test and one-way ANOVA by SigmaPlot 11.0. Post hoc Bonferroni test was used for further comparison. If the data did not pass the equal variance test, one way ANOVA was done in ranks and Dunn’s method was used for post-hoc test. Two-way ANOVA and Post hoc Bonferroni test was performed in the neuromodulator experiment to compare layer difference. In all cases, statistical significance was accepted at the *P* < 0.05 significance level.

## Results

### N-type VGCCs in ACC excitatory synaptic transmission

**Figure 1:**
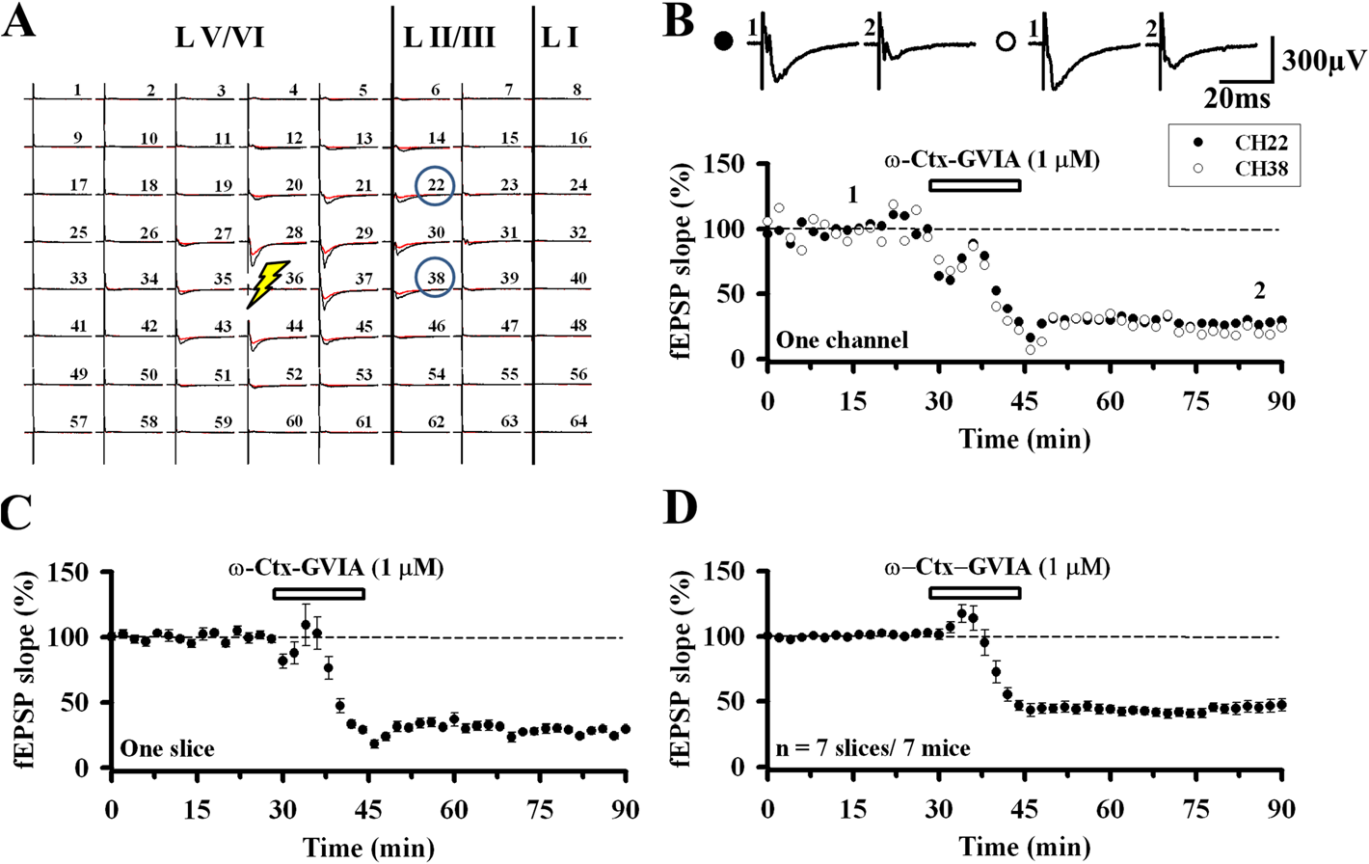
**Role of N-type VGCCs in ACC excitatory synaptic transmission in the superficial layer. A**, A representative example of MED64 responses before (black) and after (red) ω-Ctx GVIA (1 μM) application. Stimulation (thunderbolt) was given in the deep layer (Ch. 36). The regions are divided into Layer (L) I, II/III and V/VI (vertical lines). Two channels (Ch. 22 and Ch. 38) are selected for further comparison. **B**, ω-Ctx GVIA (1 μM) was applied for 15 min (filled circle: Ch. 22; open circle: Ch. 38) in a single slice. Sample fEPSP recordings taken at the times indicated by the corresponding numbers are shown above the plot. Calibration: 300 μV, 20 ms. **C**, Averaged data of 5 activated channels in the superficial layer in one slice. **D**, Pooled data of 7 mice (45.8 ± 4.4% of baseline, n = 7 slices/7 mice). Application of ω-Ctx GVIA produced an irreversible inhibition of synaptic responses recorded from the superficial layer of the ACC. The horizontal bars indicate the period of drug application. Error bars represent SEM.

In the present study, a multi-channel recording system, MED64, was used throughout the experiment to sort out the layer-related differences in synaptic responses within the ACC of adult mice. An ACC slice was placed on top of the 8 × 8 square shaped MED64 probe as previously reported [24,30]. We stimulated one channel in the deep layer V and observed widespread responses throughout the layers except layer I (Figure 1A). In the superficial layer, especially layer II/III, most of the channels showed a great reduction after bath application of the N-type VGCC blocker, ω-Ctx GVIA (1 μM, 15 min). For example, the two channels within the superficial layer (Ch. 22 and 38) both underwent similar amount of reduction (39.2% of baseline and 38.2% of baseline, Figure 1B). The effect of the drug was irreversible, with the reduction not recovered until 1 h after the onset of the drug application. The averaged fEPSP slope of 5 activated channels in the superficial layer is similar to the result of one single channel (Figure 1C). Thus, there are no apparent differences among the channels within the same layer. The summarized data show that the fEPSP slope was inhibited to 45.8 ± 4.4% of baseline after applying ω-Ctx GVIA (n = 7 slices/7 mice; Figure 1D).

**Figure 2:**
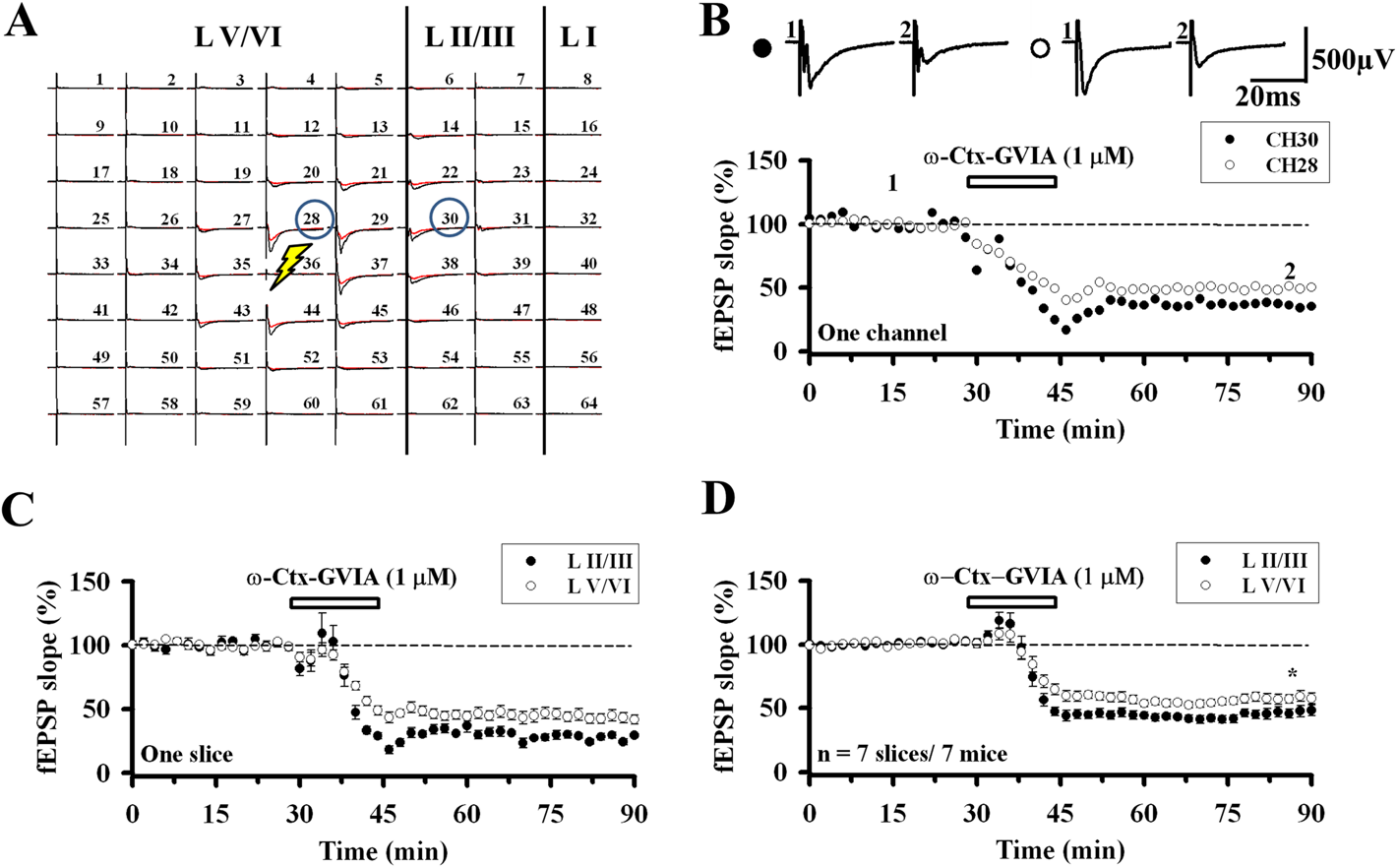
**Comparison of the effects of the N-type VGCC blocker in the superficial layer and deep layer of the ACC. A**, The same slice as Figure 1A. Two channels (Ch. 30 from LII/III and Ch. 28 from LV/VI) are selected for further comparison. **B**, ω-Ctx GVIA (1 μM) was bath applied for 15 min (filled circle: Ch. 30; open circle: Ch. 28) in a single slice. Sample fEPSP recordings taken at the times indicated by the corresponding numbers are shown above the plot. Calibration: 500 μV, 20 ms. **C**, Averaged data of 5–7 activated channels in the superficial layer (filled circle: LII/III) and deep layer (open circle: LV/VI) in one slice. **D**, Pooled data of 7 mice (LII/III: 45.8 ± 4.4% of baseline, LV/VI: 59.6 ± 2.8% of baseline, n = 7 slices/7 mice, *t*_(12)_ = −2.663, *P* = 0.021). ω-Ctx GVIA produced a stronger inhibition of superficial layer responses than those of the deep layer in the ACC. The horizontal bars indicate the period of drug application. Asterisk indicates the statistical significance between superficial layer and deep layer of the ACC slice. Error bars represent SEM.

To determine if the contribution of the N-type calcium channel to synaptic transmission varies according to the cortical layers, we compared the effect of ω-Ctx GVIA in different layers in the same slice. We selected two channels (Ch. 30 and 28) that had similar response size and shape in superficial and deep layer (layer V/VI) of the ACC, respectively (Figure 2A). Significant difference was detected in the reduction process and the inhibited level between the two channels after drug treatment (Ch. 30: 37.2% of baseline; Ch. 28: 51.2% of baseline; Figure 2B). The averaged data of 5–7 channels for one single slice (Figure 2C) and the pooled data of several mice demonstrate the same layer-related difference (superficial layer: 45.8 ± 4.4% of baseline, deep layer: 59.6 ± 2.8% of baseline, n = 7 slices/7 mice, *t*_(12)_ = −2.663, *P* = 0.021; Figure 2D). These results suggest that N-type VGCCs mediate excitatory synaptic transmission in the ACC and there were differences between layers.

### Role of other VGCCs in ACC synaptic transmission

**Figure 3:**
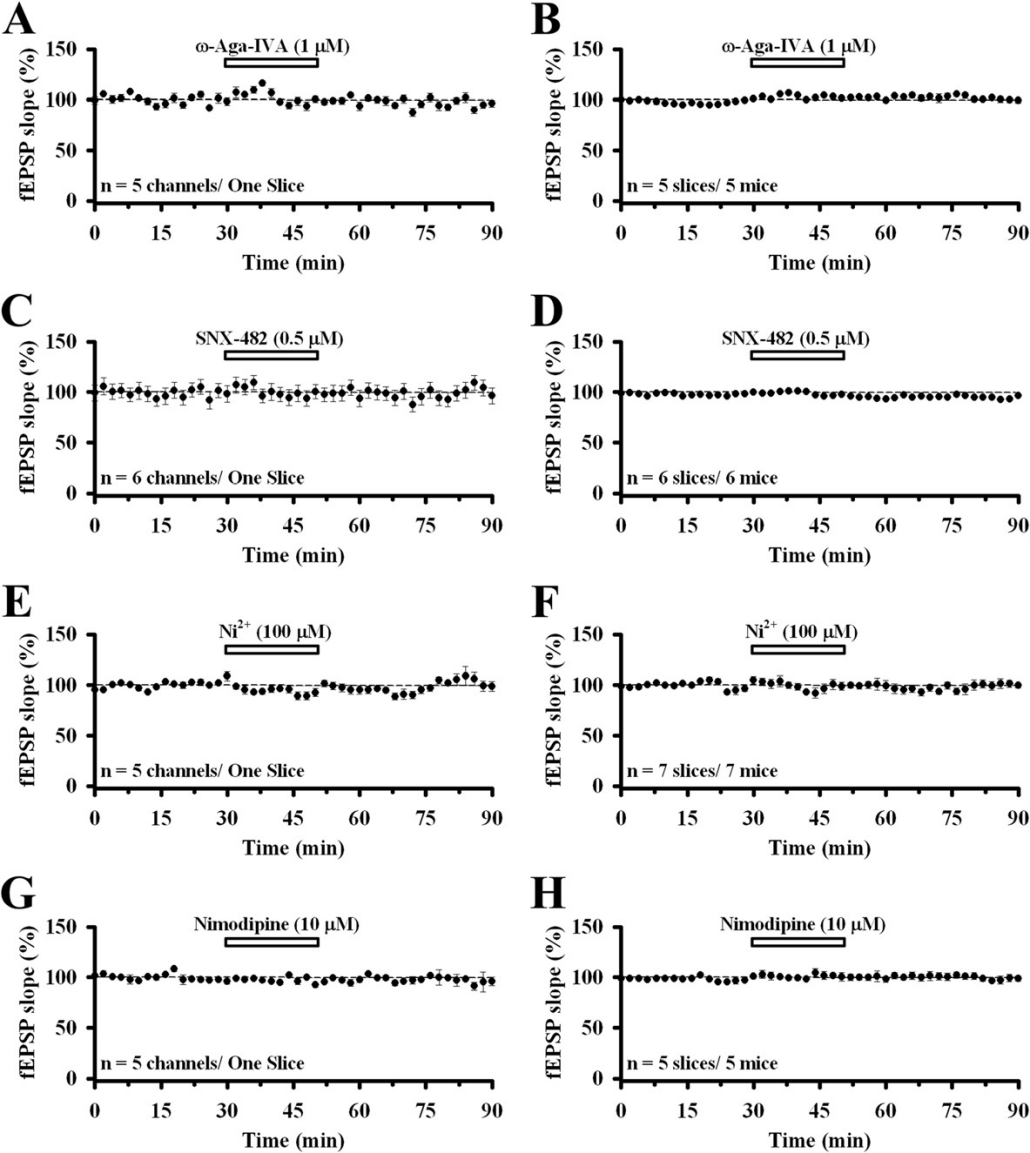
**Role of other VGCCs in ACC excitatory synaptic transmission in the superficial layer. A**, Averaged data of 5 channels in the superficial layer in one slice, showing the effect of applying ω-Aga IVA (1 μM) for 20 min. **B**, Pooled data of 5 mice (99.5 ± 1.8% of baseline, n = 5 slices/5 mice). **C**, Averaged data of 6 channels in the superficial layer in one slice, showing the effect of applying SNX-482 (0.5 μM) for 20 min. **D**, Pooled data of 6 mice (97.5 ± 0.8% of baseline, n = 6 slices/6 mice). **E**, Averaged data of 5 channels in the superficial layer in one slice, showing the effect of applying NiCl_2_ (100 μM) for 20 min. **F**, Pooled data of 7 mice (97.8 ± 2.2% of baseline, n = 7 slices/7 mice). **G**, Averaged data of 5 channels in the superficial layer in one slice, showing the effect of applying nimodipine (10 μM) for 20 min. **H**, Pooled data of 5 mice (99.2 ± 2.3% of baseline, n = 5 slices/5 mice). All drugs infused had no effect on synaptic transmission in the superficial layer of the ACC. The horizontal bars indicate the period of drug application. Error bars represent SEM.

We next tested the effects of other VGCC blockers in ACC glutamatergic synaptic transmission in the superficial layer. The P/Q-type VGCC blocker ω-Aga IVA (1 μM, 20 min) had no effect at all (Figure 3A). The level of response was 103.0 ± 1.4% at the end of the drug treatment and 99.5 ± 1.8% at the end of the recording (n = 5 slices/5 mice; Figure 3B). This is different from previous reports of the predominant role of P/Q-type VGCC in excitatory synaptic transmission in the hippocampus [2,4,6] and cerebellum [3]. R-type VGCC has been suggested to contribute to excitatory synaptic transmission in the rat hippocampus [38]. We addressed the involvement of R-type calcium channel by bath applying SNX-482 (0.5 μM, 20 min). We found that SNX-482 did not produce any significant effect (end of drug: 96.7 ± 1.4%, end of experiment: 97.5 ± 0.8%, n = 6 slices/6 mice; Figure 3C and D). Next, we tested the possible contribution of low-voltage activated T-type VGCC by applying the T-type VGCC blocker NiCl_2_ (100 μM, 20 min). We found that there was no change of the synaptic transmission in the superficial layer (end of drug: 98.8 ± 4.4%, end of experiment: 97.8 ± 2.2%, n = 7 slices/7 mice; Figure 3E and F). Finally, we tested if the L-type VGCC mediates the ACC synaptic transmission. Bath infusion of nimodipine (10 μM, 20 min) failed to produce any inhibitory effect on the slope of fEPSP recorded from the superficial layer of the ACC slice (end of drug: 101.2 ± 3.3%, end of experiment: 99.2 ± 2.3%, n = 5 slices/5 mice; Figure 3G and H).

**Figure 4:**
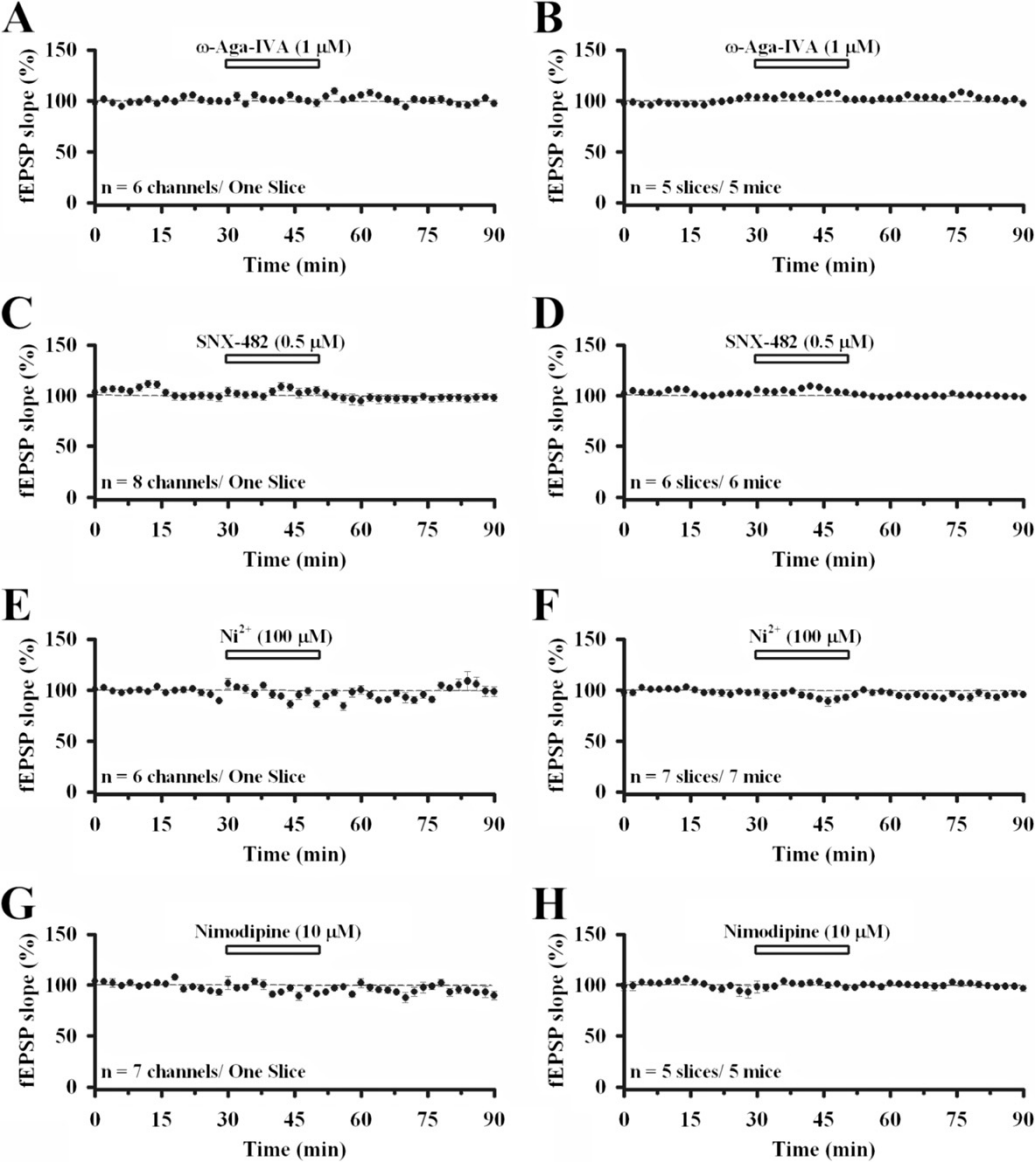
**Role of other VGCCs in ACC excitatory synaptic transmission in the deep layer. A**, Averaged data of 6 channels in the deep layer in one slice, showing the effect of applying ω-Aga IVA (1 μM) for 20 min. **B**, Pooled data of 5 mice (96.9 ± 1.4% of baseline, n = 5 slices/5 mice). **C**, Averaged data of 8 channels in the deep layer in one slice, showing the effect of applying SNX-482 (0.5 μM) for 20 min. **D**, Pooled data of 6 mice (98.0 ± 0.2% of baseline, n = 6 slices/6 mice). **E**, Averaged data of 6 channels in the deep layer in one slice, showing the effect of applying NiCl_2_ (100 μM) for 20 min. **F**, Pooled data of 7 mice (97.7 ± 1.3% of baseline, n = 7 slices/7 mice). **G**, Averaged data of 7 channels in the deep layer in one slice, showing the effect of applying nimodipine (10 μM) for 20 min. **H**, Pooled data of 5 mice (98.0 ± 1.9% of baseline, n = 5 slices/5 mice). All drugs infused had no effect on synaptic transmission in the deep layer of the ACC. The horizontal bars indicate the period of drug application. Error bars represent SEM.

Similar results were found in the deep layer of the ACC. The ω-Aga IVA group showed 105.2 ± 1.4% at the end of drug treatment and 96.9 ± 1.4% at the end of experiment (n = 5 slices/5 mice; Figure 4A and B). SNX-482 (end of drug: 104.1 ± 0.7%, end of experiment: 98.0 ± 0.2%, n = 6 slices/6 mice; Figure 4C and D), NiCl_2_ (end of drug: 91.2 ± 3.2%, end of experiment: 97.7 ± 1.3%, n = 7 slices/7 mice; Figure 4E and F) and nimodipine (end of drug: 99.3 ± 2.1%, end of experiment: 98.0 ± 1.9%, n = 5 slices/5 mice; Figure 4G and H) all showed no inhibition.

**Figure 5:**
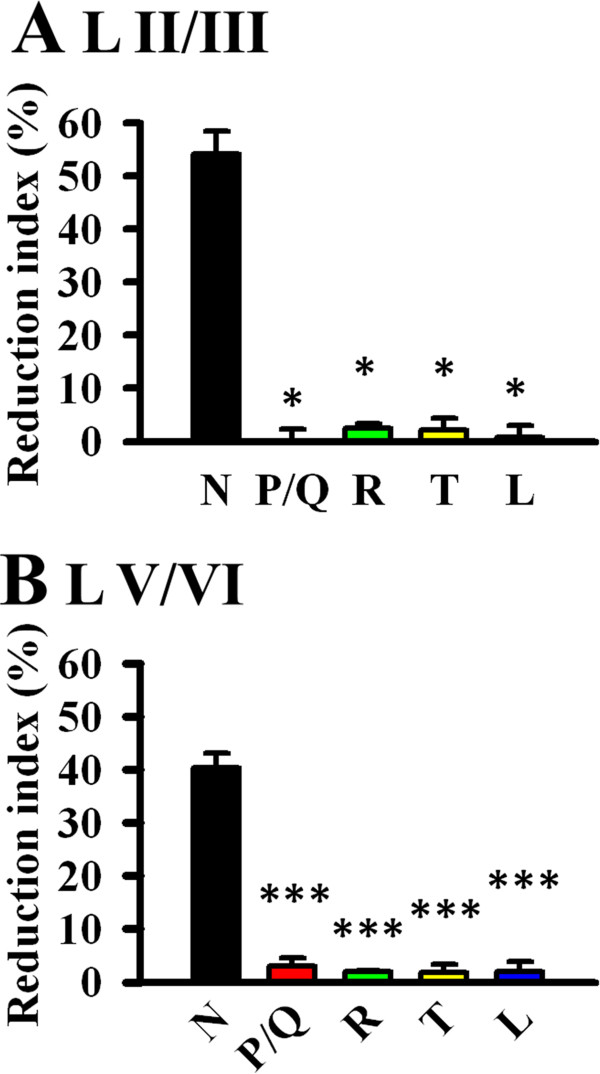
**The predominant role of N-type VGCC in ACC excitatory synaptic transmission. A**, The difference in the reduction index (100% - the normalized fEPSP slope value of the last 4 min of the experiment) among the drug treatment groups in the superficial layer is statistically significant (*F*_(4,25)_ = 16.00, *P* = 0.003, One-way ANOVA in ranks with Dunn’s post-hoc; *P* < 0.05 between N-type and all other groups). **B**, The difference in the deep layer is also statistically significant (*F*_(4,25)_ = 93.53, *P* < 0.001, One-way ANOVA with Bonferroni’s post-hoc; *P* < 0.001 between N-type and all other groups). Error bars represent SEM.

The difference in the reduction index among the drug treatment groups in the superficial layer was statistically significant (*F*_(4,25)_ = 16.00, *P* = 0.003, One-way ANOVA in ranks with Dunn’s post-hoc; *P* < 0.05 between N-type and all other groups, Figure 5A). There was also significant difference in the deep layer (*F*_(4,25)_ = 93.53, *P* < 0.001, One-way ANOVA with Bonferroni’s post-hoc; *P* < 0.001 between N-type and all other groups, Figure 5B). Overall, N-type is the predominant VGCC involved in excitatory synaptic transmission in the ACC. Other VGCCs we tested do not participate in the ACC glutamatergic synaptic transmission.

### Modulation of ACC synaptic transmission

**Figure 6:**
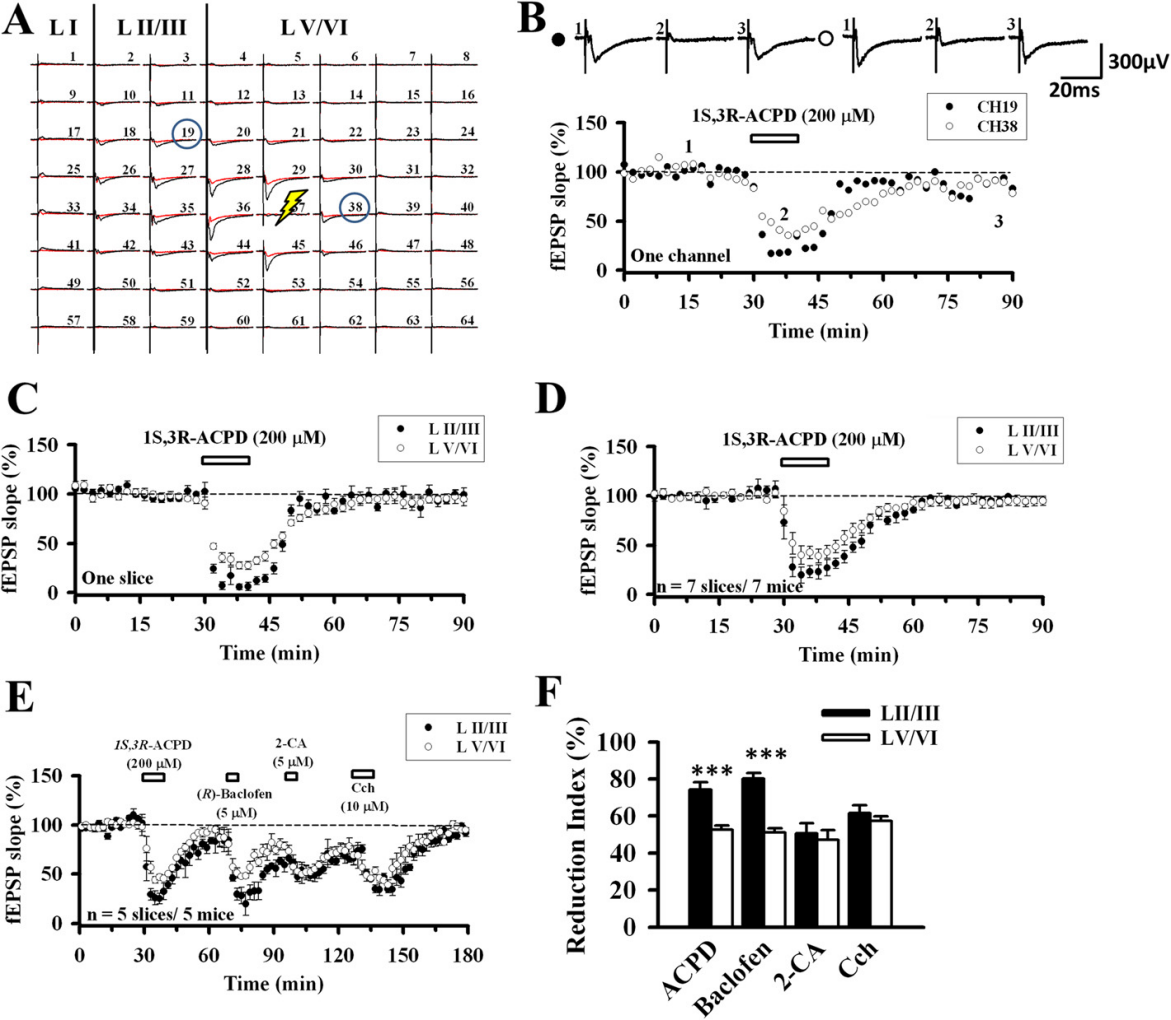
**Effect of neuromodulators on ACC excitatory synaptic transmission. A**, A representative example of MED64 responses before (black) and after (red) 1*S*,3*R*–ACPD (200 μM) application. Stimulation (thunderbolt) was given in the deep layer (Ch. 37). Two channels (Ch. 19 from LII/III and Ch. 38 from LV/VI) are selected for further comparison. **B**, 1*S*,3*R*–ACPD (200 μM) was bath applied for 10 min (filled circle: Ch. 19; open circle: Ch. 38) in a single slice. Sample fEPSP recordings taken at the times indicated by the corresponding numbers are shown above the plot. Calibration: 300 μV, 20 ms. **C**, Averaged data of 5–7 activated channels in the superficial layer and deep layer in one slice. **D**, Pooled data of 7 mice (LII/III: 23.2 ± 6.5% of baseline, LV/VI: 42.7 ± 5.5% of baseline, n = 7 slices/7 mice). Bath application of 1*S*,3*R*-ACPD resulted in an acute inhibition that gradually recovered after washout. The superficial layer response exhibited a much more reduction than the deep layer. **E**, Four neuromodulators were sequentially applied. Significant differences could be found in the blocking effect of 1S,3R–ACPD and (*R*)-Baclofen between individual layers (n = 5 slices/5 mice). The horizontal bars indicate the period of drug application. Error bars represent SEM. **F**, Reduction index of the neuromodulators. 1*S*,3*R*–ACPD (LII/III: 74.3% ± 8.8%, LV/VI: 52.5 ± 2.3%, *P* = 0.003) and (*R*)-Baclofen (LII/III: 80.3 ± 2.8%, LV/VI: 51.1 ± 2.2%, *P* = 0.002) showed statistically significant difference between the layers. 2-CA (LII/III: 50.6 ± 5.5%, LV/VI: 47.1 ± 5.2%, *P* = 0.614) and Cch (LII/III: 61.5 ± 4.2%, LV/VI: 57.3 ± 2.5%, *P* = 0.538) exhibited no layer-related difference.

We next investigated whether the ACC synaptic transmission is responsive to the modulation exerted by several neuromodulators in the brain. Neuromodulators are signals that trigger activation or inhibition of the neurons and many of them are known to affect VGCCs [4,39-41]. Because only the N-type VGCC blocker showed a significant reduction in the ACC synaptic transmission, we decided to apply several neuromodulators with or without the presence of ω-Ctx GVIA (1 μM). 1*S*,3*R*–ACPD (200 μM, 10 min), an mGluR agonist, was first tested without applying ω-Ctx GVIA (Figure 6A). One channel in the superficial layer (Ch. 19) and the other in the deep layer (Ch. 38) were selected to compare its effect. The drug induced a great inhibition during the treatment and after washout the response recovered to the baseline level in both superficial and deep layers. The reduction extent of deep layer during drug application was less than that of the superficial layer (Ch. 19: 22.0% of baseline; Ch. 38: 44.6% of baseline, Figure 6B). This trend was also seen in the averaged data of 5–7 channels in one slice (Figure 6C). Pooled data from 7 animals show the same layer-related difference (LII/III: 23.2 ± 6.5%, LV/VI: 42.7 ± 5.5%, n = 7 slices/7 mice, *t*_(8)_ = −2.280, *P* = 0.042; Figure 6D).

To compare the inhibitory effects among different neuromodulators, we sequentially applied several drugs to the same slice. After the application of 1*S*,3*R*–ACPD (200 μM) for 10 min, we then applied GABA_B_ receptor agonist (*R*)-Baclofen (5 μM) for 5 min, adenosine receptor agonist 2-CA (5 μM) for 5 min and finally cholinergic receptor agonist carbachol (Cch, 10 μM) for 10 min (Figure 6E). In a previous study of the hippocampus, these drugs all inhibited the synaptic transmission immediately after they were applied with full recovery after washout [4]. We found similar results in the ACC slices but the recovery was partial after the washout. In the superficial layer, 1*S*,3*R*–ACPD inhibited the response to 25.7 ± 3.9% of baseline, (*R*)-Baclofen to 19.7 ± 2.8%, 2-CA to 49.4 ± 5.5%, and Cch to 38.5 ± 4.2% (n = 5 slices/5 mice). The magnitude of inhibition induced by the drugs in the deep layer was: 1*S*,3*R*–ACPD 47.5 ± 2.3%, (*R*)-Baclofen 48.8 ± 2.2%, 2-CA 52.9 ± 5.2% and Cch 42.6 ± 2.5% (n = 5 slices/5 mice). Generally, there was statistically significant difference between layers (*F*_(1,8)_ = 6.427, *P* = 0.035, Repeated two-way ANOVA). Post hoc Bonferroni analysis revealed statistical differences between LII/III and LV/VI for 1*S*,3*R*–ACPD (*P* = 0.013) and (*R*)-Baclofen (*P* < 0.001). 2-CA (*P* = 0.673) and Cch (*P* = 0.714) showed no layer-related difference (see Figure 6E). Analysis of the reduction index obtained the same results (Figure 6F). Both 1*S*,3*R*–ACPD and (*R*)-Baclofen produced a much stronger inhibition in the superficial layer than in the deep layer (*P* < 0.001 for both drugs).

**Figure 7:**
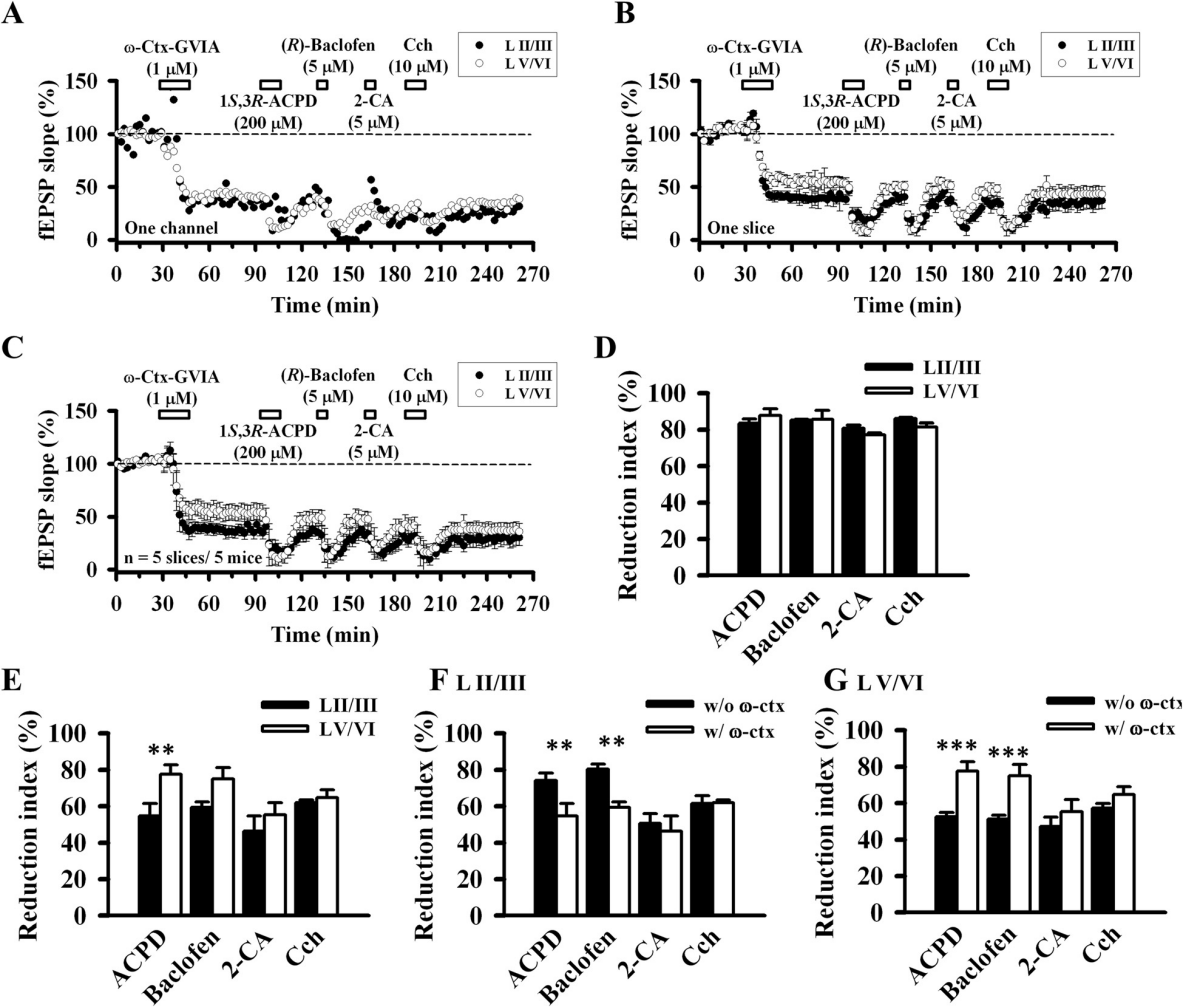
**Effect of neuromodulators after blocking N-type VGCC. A**, Results of one channel applying each drug sequentially (filled circle: LII/III, open circle: LV/VI): ω-Ctx-GVIA (1 μM) for 15 min, 1*S*,3*R*–ACPD (200 μM) for 10 min, (*R*)-Baclofen (5 μM) for 5 min, 2-CA (5 μM) for 5 min and Cch (10 μM) for 10 min. **B**, Averaged data (n = 6–8 channels/1 slice) of each layer that showed response in one slice. **C**, Pooled data of 5 mice. Pre-exposure of the ACC slice with ω-Ctx-GVIA abolished the layer-related difference in the effect of 1*S*,3*R*–ACPD and (*R*)-Baclofen (n = 5 slices/5 mice). The horizontal bars indicate the period of drug application. Error bars represent SEM. **D**, Reduction index of ω-Ctx-GVIA and neuromodulators in different layers. All drugs showed no statistically significant difference between layers. **E**, Reduction index of neuromodulators when ω-Ctx-GVIA-insensitive responses (70–90 min in Figure 7C) were normalized as 100%. 1*S*,3*R*–ACPD inhibited the ω-Ctx GVIA-insensitive responses more in the deep layer (LII/III: 54.6 ± 7.0%, LV/VI: 77.7 ± 5.0%, *P* = 0.007). The layer difference was not clear for Baclofen (LII/III: 59.3 ± 2.9%, LV/VI: 75.0 ± 6.3%, *P* = 0.057), 2-CA (LII/III: 46.3 ± 8.3%, LV/VI: 55.4 ± 6.5%, *P* = 0.262) and Cch (LII/III: 61.9 ± 1.5%, LV/VI: 64.8 ± 4.3%, *P* = 0.719). **F**, Reduction index of each agonist in the superficial layer with (Figure 6F) or without ω-Ctx GVIA (Figure 7E). **G**, Reduction index of each agonist in the deep layer with (Figure 6F) or without ω-Ctx GVIA (Figure 7E).

We next checked whether these effects will change after applying ω-Ctx GVIA (1 μM, 15 min) to block N-type VGCC-mediated synaptic transmission (Figure 7A-C). Similarly, ω-Ctx GVIA induced irreversible synaptic inhibition even after washout. We then applied the neuromodulator as Figure 6E. All the drugs still produced inhibition of the fEPSP recorded from the ACC. The inhibited values in the superficial layer were: 1*S*,3*R*–ACPD 16.7 ± 2.4%, (*R*)-Baclofen 14.8 ± 0.3%, 2-CA 19.2 ± 1.9%, Cch 14.0 ± 0.7% (n = 5 slices/5 mice, Figure 7C). In the deep layer, the values were: 1*S*,3*R*–ACPD 12.3 ± 3.7%, (*R*)-Baclofen 14.4 ± 4.8%, 2-CA 22.8 ± 1.1%, Cch 18.5 ± 2.1% (n = 5 slices/5 mice, Figure 7C). Repeated two-way ANOVA detected the statistically significant differences between layers (*F*_(1,8)_ = 7.069, *P* = 0029). However, post doc Bonferroni test failed to show any layer-related significant change for all of the four drugs (Figure 7D). Thus, although the N-type VGCC blocker did not prevent neuromodulators-induced synaptic inhibition, it abolished the layer difference in the effects of 1*S*,3*R*–ACPD and (*R*)-Baclofen.

To further elucidate the mechanisms for this loss of layer difference, we normalized the 70–90 min period of Figure 7C as 100% and checked the reduction index of the neuromodulators again (Figure 7E). We found that 1*S*,3*R*–ACPD inhibited the ω-Ctx GVIA-insensitive responses from the deep layer to a greater extent than those in the superficial layer (LII/III: 54.6 ± 7.0%, LV/VI: 77.7 ± 5.0%, *P* = 0.007). The layer difference was not clear for Baclofen (LII/III: 59.3 ± 2.9%, LV/VI: 75.0 ± 6.3%, *P* = 0.057), 2-CA (LII/III: 46.3 ± 8.3%, LV/VI: 55.4 ± 6.5%, *P* = 0.262) and Cch (LII/III: 61.9 ± 1.5%, LV/VI: 64.8 ± 4.3%, *P* = 0.719, Figure 7E). Comparison of the reduction index with and without the presence of ω-Ctx GVIA reached the same conclusion for the layer difference (Figure 7F and G). These data suggest that 1*S*,3*R*–ACPD possibly acts more in the deep layer with different mechanisms when the N-type VGCC was blocked.

### Whole-cell patch-clamp recordings of synaptic transmission in the ACC

**Figure 8:**
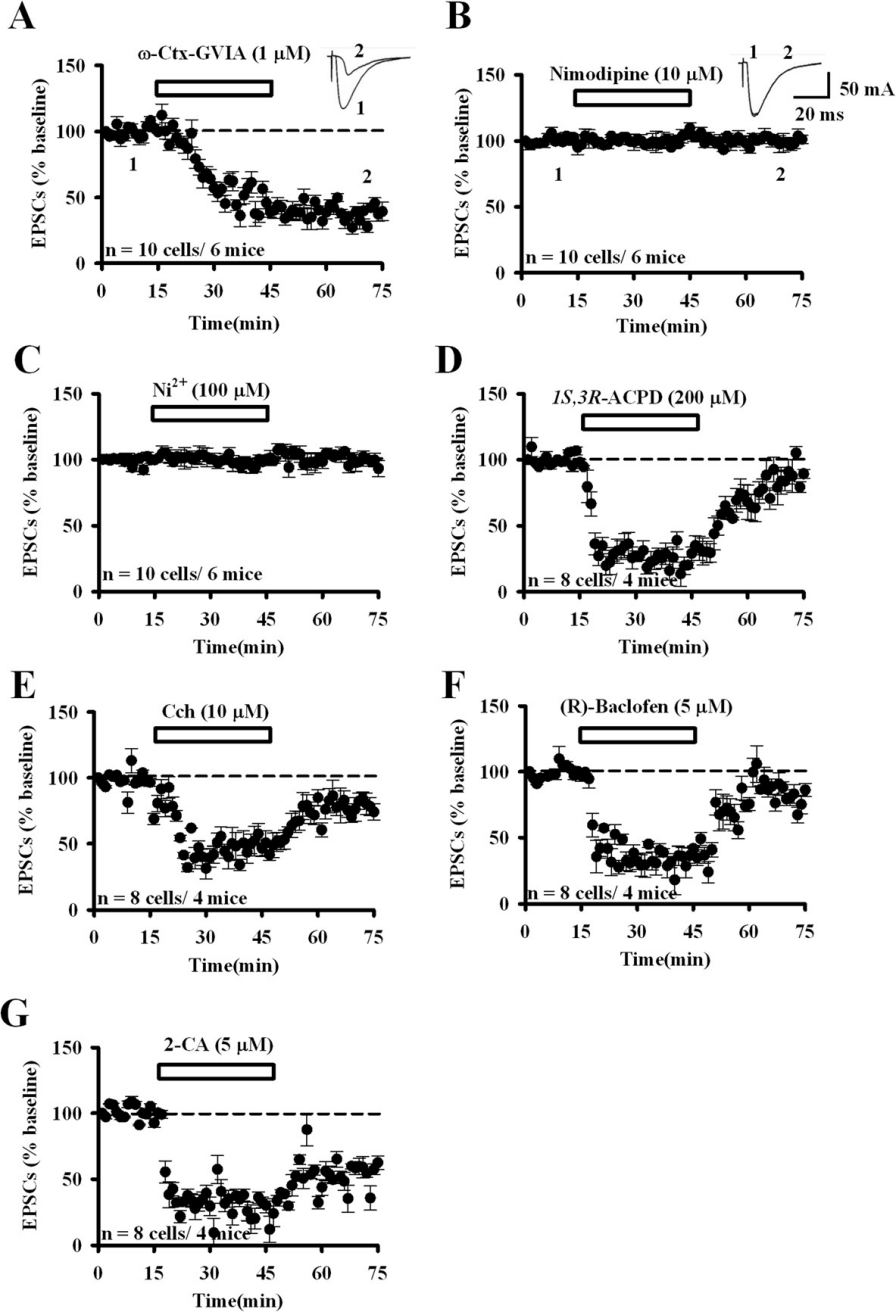
**Whole-cell patch-clamp recordings of the synaptic responses in the ACC: N-type VGCC involvement and modulation by neuromodulators. A**, ω-Ctx-GVIA (1 μM, 30 min) treatment blocked the EPSCs recorded from LII/III neurons of the ACC (39.2 ± 7.0%, n = 10 cells/6 mice). Sample traces are shown in the insets. **B**, **C**, Nimodipine (10 μM, 30 min, 101.2 ± 3.0%, n = 10 cells/6 mice) and NiCl_2_ (100 μM, 30 min, 99.6 ± 5.1%, n = 10 cells/6 mice) did not block the synaptic response. Sample traces of nimodipine treatment are shown in the insets. **D-F**, Application of 1*S*,3*R*–ACPD (200 μM, 29.1 ± 5.1%, n = 8 cells/4 mice), Cch (10 μM, 46.7 ± 6.2%, n = 8 cells/4 mice) and (*R*)-Baclofen (5 μM, 41.7 ± 2.0%, n = 8 cells/4 mice) for 30 min inhibited the ACC LII/III neuronal responses and the inhibition was recovered after washout. **G**, 2-CA (5 μM, 29.9 ± 5.3%, n = 8 cells/4 mice) induced a similar acute inhibition but the inhibition is maintained during washout. The horizontal bars indicate the period of drug application. Error bars represent SEM.

To confirm the MED64 data, we performed whole-cell patch-clamp recordings in the ACC LII/III neurons (Figure 8). Three VGCC blockers were bath infused for 30 min and only ω-Ctx GVIA showed the blocking effect in the ACC synaptic transmission (ω-Ctx GVIA: 39.2 ± 7.0% of baseline, n = 10 cells/6 mice; nimodipine: 101.2 ± 3.0% of baseline, n = 10 cells/6 mice; NiCl_2_: 99.6 ± 5.1% of baseline, n = 10 cells/6 mice; Figure 8A-C). In addition, we also applied the neuromodulators and examined their influence on the EPSCs recorded from the superficial layer of the ACC. Similarly, we found that all of the four drugs produced an acute inhibition that can be fully or partially recovered after washout (1S,3R–ACPD: 200 μM, 29.1 ± 5.1% of baseline; Cch: 10 μM, 46.7 ± 6.2% of baseline; (*R*)-Baclofen: 5 μM, 41.7 ± 2.0% of baseline; 2-CA: 5 μM, 29.9 ± 5.3% of baseline; n = 8 cells/4 mice; Figure 8D-G). Nevertheless, comparison of the whole-cell and MED64 data revealed some differences in the reduction extent and recover process of the drug effect. For example, 2-CA produced a much larger extent of reduction in EPSCs (29.9 ± 5.3% of baseline at the end of the drug treatment, n = 8 cells/4 mice, Figure 8G) as compared to its effect on fEPSPs (49.4 ± 5.5% of baseline at the end of the drug treatment, n = 5 slices/5 mice, Figure 6E). Also, while the 2-CA-induced fEPSP inhibition is fully reversible (Figure 6E), the suppression of EPSCs cannot be recovered (Figure 8G). This discrepancy may be attributed to differences in experimental variables such as the recording method (whole-cell patch-clamp recording vs. multi-channel field potential recording) and drug infusion time (30 min vs. 5 min).

## Discussion

In this study, we have demonstrated the prominent role of N-type VGCC in mediating glutamatergic synaptic transmission in the adult mice ACC. We have used the newly-developed MED64 system to record multisite synaptic responses in the coronal ACC slices and compare the possible layer-related differences. Several types of VGCC blockers were tested in the basal synaptic transmission and only ω-Ctx GVIA, the N-type VGCC blocker, showed a great reduction. Moreover, the superficial layer had a greater inhibition than the deep layer. We also tested whether ω-Ctx GVIA would influence the modulatory effect of several neuromodulators on excitatory synaptic transmission in the ACC. We found that the neuromodulator effects were not greatly affected by ω-Ctx GVIA.

### N- and P/Q-type VGCC in the ACC

N- and P/Q-type calcium channels are the major VGCCs for Ca^2+^ influx to initiate the fast release of neurotransmitters/neuromodulators such as glutamate, acetylcholine, and GABA [42]. Both channels are high-voltage activated channels consisting of the α_1_ subunit pore. N-type VGCC comprises an α_1B_ subunit or Ca_v2.2_ and the channel is primarily in the presynaptic compartment [8,18,42]. ω-Ctx GVIA was commonly used to antagonize the activity of N-type VGCC [1,4,6,18,20]. There are studies demonstrating the up-regulation of N-type VGCC subunit in the primary afferent neurons after tissue inflammation and nerve injury [12,43,44]. There is an N-type VGCC-targeting drug (Ziconotide) used in the clinic to relieve neuropathic and inflammation pain. However, its clinical application is limited by the accompanying central side effects and can only be applied spinally [18-21]. Our studies provide strong evidence that N-type calcium channels play important roles in the ACC synaptic transmission, and the inhibitory effect of the N-type VGCC blocker in the ACC may explain some of the side effects caused by Ziconotide.

P/Q-type VGCC consists of an α_1A_ subunit or Ca_v2.1_ and ω-Aga IVA is the well-known antagonist. Several studies show that dysfunction of this channel induces ataxia, migraine, vertigo and epilepsy [45-48]. The involvement of P/Q-type VGCC in central synaptic transmission and plasticity has been extensively studied in the hippocampus [2,4,6], cerebellum [3] and nucleus accumbens [49]. However, there are no studies of N- and P/Q-type VGCC engagement in the adult mice ACC. Our results revealed a dramatic reduction of glutamatergic synaptic transmission by the N-type VGCC blocker in the ACC. Since the excitatory synaptic responses in the ACC are mediated predominantly by AMPA/kainate receptors, the explanation for ω-Ctx GVIA-induced inhibition would be the reduction of presynaptic glutamate release [30]. Interestingly, ω-Ctx GVIA produced a much stronger inhibition of the superficial layer responses than those of the deep layer, indicating that the expression density of the N-type VGCC in the ACC may have layer-related difference. Future studies are required to address this issue by using morphological tools to investigate the distribution of VGCCs across each layer of the ACC.

The current findings show other VGCC blockers did not significantly inhibit ACC synaptic response. These results differ from previous reports in other brain regions, which involve the combination of N- and P/Q-type VGCCs in excitatory synaptic transmission [9,10]. The possibility of low doses of the drugs causing the negative results is unlikely, because the drug concentrations used in the present experiment are not lower compared to other studies [4,10,49]. According to the *in situ* hybridization data from Allen Institute for Brain Sciences, all types of VGCCs are expressed in the ACC. Therefore, they may exert some other functions rather than mediating the excitatory synaptic transmission. For example, it has been shown in our previous studies that L-type VGCC is involved in low frequency stimulation-induced long-term depression in the ACC [30,50]. Taken together, these findings indicate that different central synapses in the brain may depend on different types of VGCCs for mediation of the excitatory synaptic transmission. Notably, however, ω-Ctx GVIA only blocked about 50% of the total synaptic transmission in the ACC. Therefore, it is still necessary for future studies to identify the receptors or channels mediating the remaining 50% of the ACC synaptic response in the basal condition.

### VGCCs and neuromodulators in the ACC

N-, P/Q-, R-, and L- type VGCCs are all involved in presynaptic neurotransmitter release [9,42] and it has been reported that different neuromodulators affect these VGCCs [4,39-41]. Thus, we wanted to test the effect of ω-Ctx GVIA on the modulation of ACC synaptic transmission exerted by various neuromodulators. We have applied four neuromodulators with or without the presence of ω-Ctx GVIA. All drugs induced a great acute inhibition of the fEPSP under both conditions and the inhibitive effect is reversible after washout. These results are partially consistent with the previous reports in the hippocampus [4]. Interestingly, the layer-related difference regarding the inhibitory effect of 1S,3R–ACPD and (R)-Baclofen is abolished after blocking N-type VGCC-mediated synaptic transmission. One possible explanation for layer-related difference is different distribution of mGluRs (for 1S,3R–ACPD) and GABA_B_ (for (R)-Baclofen) among synapses in the superficial vs deep layers of the ACC. Future studies are needed to further investigate these mechanisms via a combination of electrophysiological and morphological approaches.

The modulation of synaptic transmission by 1S,3R–ACPD in the ACC is consistent with the previous reports in other brain areas, such as hippocampus [4,51-53], neocortex [54], cerebellum [55] and striatum [56]. These data are also consistent with the previous results showing the inhibition of N-type and other types of VGCC currents by the 1S,3R-ACPD [57-63]. It is likely that 1S,3R-ACPD produces its inhibitory effect by intracellular G protein coupled signaling pathways. Considering the fact that 1S,3R-ACPD is a non-selective mGluR antagonist [64,65], future studies are needed to examine the subtypes of mGluRs and their related downstream signaling pathways mediating the inhibitory effects.

In summary, our present study is the first to establish the importance of N-type VGCC in mediating excitatory synaptic transmission in the adult mice ACC. ACC is known to be an important region for memory and chronic pain [22,23]. Due to its important roles in normal synaptic transmission in the ACC, our results provide possible explanations for the central side effects produced by the N-type calcium channel blockers applied intrathecally or systemically in the clinic.

## Abbreviations

1S,3R-ACPD: (1S,3R)-1-aminocyclopentane-1,3-dicarboxylic acid; 2-CA: 2-Chloroadenosine; ACC: Anterior cingulate cortex; ACSF: Artificial cerebrospinal fluid; Cch: Carbachol; EPSC: Excitatory postsynaptic current; fEPSP: Field excitatory postsynaptic potential; GABAB: γ-Aminobutyric acid B; MED64: 64-channel multi-electrode dish; mGluR: Metabotropic glutamate receptor; VGCC: Voltage-gated calcium channel; ω-Ctx GVIA: ω-conotoxin GVIA; ω-Aga IVA: ω-agatoxin IVA.

## Competing interests

The authors declare that they have no competing interests.

## Authors’ contributions

SJK designed, performed the experiments and wrote the manuscript. MGL performed some of the MED64 experiments and revised the manuscript. TYS performed the whole-cell patch-clamp recordings. MGZ and BKK supervised the experiments and commented on the manuscript. MZ participated in the experiment design and coordination, and helped to finish the manuscript. All authors read and approved the final manuscript.
